# Quantitative analysis of bone morphogenetic protein 15 (BMP15) and growth differentiation factor 9 (GDF9) gene expression in calf and adult bovine ovaries

**DOI:** 10.1186/1477-7827-9-33

**Published:** 2011-03-15

**Authors:** Misa Hosoe, Kanako Kaneyama, Koichi Ushizawa, Ken-go Hayashi, Toru Takahashi

**Affiliations:** 1 Reproductive Biology Research Unit, Division of Animal Sciences, National Institute of Agrobiological Sciences, 2-1-2 Kannondai, Tsukuba, Ibaraki 305-8602, Japan; 2 Department of Technology, National Livestock Breeding Center, 1 Odakurahara, Odakura, Nishigo, Fukushima 961-8511, Japan

## Abstract

**Background:**

It has been reported that calf oocytes are less developmentally competent than oocytes obtained from adult cows. Bone morphogenetic protein 15 (BMP15) and growth and differentiation factor 9 (GDF9) play critical roles in folliculogenesis, follicular development and ovulation in mammalian ovaries. In the present study, we attempted to compare the expression patterns of *BMP15 *and *GDF9 *in the cells of calf and cow ovaries to determine a relationship between the level of these genes and the low developmental competence of calf oocytes.

**Methods:**

Bovine tissues were collected from 9-11 months-old calves and from 4-6 years-old cows. We characterized the gene expression of BMP15 and GDF9 in calf and adult bovine oocytes and cumulus cells using quantitative real-time reverse transcriptase polymerase chain reaction (QPCR) and *in situ *hybridization. Immunohistochemical analysis was also performed.

**Results:**

The expression of *BMP15 *and *GDF9 *in cumulus cells of adult ovaries was significantly higher than that in calf ovaries, as revealed by QPCR. *GDF9 *expression in the oocytes of calf ovaries was significantly higher than in those of the adult ovaries. In contrast, *BMP15 *expression in the oocytes of calf and adult ovaries was not significantly different. The localization of gene expression and protein were ascertained by histochemistry.

**Conclusions:**

Our result showed for the first time BMP15 and GDF9 expression in bovine cumulus cells. *BMP15 *and *GDF9 *mRNA expression in oocytes and cumulus cells was different in calves and cows.

## Background

Growth and differentiation factor 9 (GDF9) and GDF9B, also known as BMP15, are members of the TGFβ superfamily [1-6]. In rodent species, BMP15 and GDF9 play a crucial role in ovarian follicular development. The two factors are thought to affect granulosa cell proliferation independently or synergistically [2,4,7] and to regulate cumulus cell function in the periovulatory period [8-10]. BMP15 inhibits FSH-induced granulosa cell differentiation through down-regulation of FSH receptor (FSHR) expression in rat granulosa cells [11]. *GDF9 *homozygous knockout (*GDF9-/-*) female mice are sterile due to the abolishment of folliculogenesis beyond the primary follicle stage [12,13]. However, such a dramatic effect is not observed in *BMP15 *homozygous knockout (*BMP15-/-*) mice [14]. The functionality of BMP15 and GDF9 in ruminants was mainly reported in sheep. Some sheep breeds, having mutation in the BMP15 and GDF9 signaling systems, have been established as valuable genetic resources for sheep farming because of their prolificacy phenotype. These breeds have a higher ovulation quota and produce more offspring than other conventional breeds [15-17].

It was reported that BMP15 and GDF9 expressed only in oocytes in rodents, but expressed in cumulus and mural granulosa cells as well as in oocytes in goats and pigs [1,3,5]. In bovine oocytes, although it has been reported that *BMP15 *and *GDF9 *mRNAs were expressed from respective primary and primordial follicles, and that the expression lasted to the 8-cell stage after fertilization [18], there is not enough information about the detailed expression profiles of BMP15 and GDF9. Recently, Hussein et al. reported that the addition of exogenous GDF9 and BMP15 to maturing cumulus-oocyte complexes (COCs) dramatically increased the yield of blastocysts [19]. They also reported that the addition of GDF9 and/or BMP15 antagonist to maturing COCs significantly decreased blastocyst yields, compared to untreated COCs [19].

In the bovine ovary, antral follicles appeared for the first time in the fetuses with a head-rear length of 70-80 cm (7-8 months pregnant) [20]. The use of newborns and prepubertal calves as oocyte donors for IVP shortens the interval between generations and prolongs the reproduction period. However, it is known that prepubertal calf oocytes are less developmentally competent than oocytes obtained from cows. Although the rates of fertilization and cleavage in prepubertal calf oocytes compared favorably with those in cow oocytes, their capacity to develop to the blastocyst stage is relatively lower [21-23]. It would seem that embryos from calf oocytes are less capable of establishing pregnancies [24]. Calf oocytes were found to be more sensitive to freezing injury than cow oocytes [25]. Calf oocytes showed a delay in organelle migration, mainly cortical granules, following *in vitro *maturation, as well as abnormal chromatin and microtubule configurations [26]. It is an important to analyze the genes that are related to the development of calf oocytes.

In this study, we characterized the gene expression of BMP15 and GDF9 in calf and adult bovine ovaries using quantitative real-time reverse transcriptase polymerase chain reaction (QPCR) and *in situ *hybridization. In addition, we also characterized the gene expression of *FSHR *in cumulus cells of calf and adult ovaries.

## Methods

### Animals and tissues

Protocols for the use of animals were approved by the Animal Care Committee of the National Institute of Agrobiological Sciences and the National Institute of Livestock and Grassland Science, Japan. Bovine tissues were collected from 9-11 months-old calves and from 4-6 years-old cows, born, grown and slaughtered at the National Institute of Livestock and Grassland Science less than 10 min after slaughtering. Tissues intended for PCR experiments were snap-frozen in liquid nitrogen and stored at -80°C. Ovaries were brought to the laboratory in PBS at 4°C and COCs were aspirated, separated into oocytes and cumulus cells and snap-frozen less than 60 min after slaughtering. Ovaries intended for histological studies were fixed with 10% formaldehyde PBS, pH 7.4 less than 10 min after slaughtering, and subsequently dehydrated, embedded in paraffin wax, and stored at 4°C.

Aspirated COCs from follicles 2-5 mm in diameter were classified according to a previous report [27]. Briefly, class A COCs have a thick cumulus layer; class B COCs have a thin cumulus layer, class C are naked oocytes, and COCs of class D have expanded cumulus cells. Oocytes, cumulus cells and mural granulosa cells from class A and B COCs were used as materials for reverse transcriptase polymerase chain reaction (RT-PCR). For RT-PCR, COCs were separated from the ovaries of calves (n = 4 animals) and cows (n = 4 animals). For *in situ *hybridization, ovaries were recovered from calves (n = 3 animals) and cows (n = 3 animals).

### RT-PCR

Liver, kidney, heart, spleen, lung, ovary and pituitary tissues were collected from the same cows. Oocytes with zona pellucida and cumulus cells in COCs were separated by vortexing and/or repeated aspiration with a narrow-bore Pasteur pipette. After collection, all cells were treated with ISOGEN (Nippon Gene, Toyama, Japan) and stored at -80°C until RNA extraction.

Total RNA extraction and reverse transcription were performed as previously reported [28]. RT-PCR was performed using two sets of primers, BMP15 (5'-CAAGCAGGCAGTATTGCATCTGAA-3' and 5'- TCACCTACATGTGCAGGACTGGGC-3'), and GDF9 (5'- AGAAGCTGCTGAGGGTGTAAGATT-3' and 5'- AAGCAATTGAGCCATCAGGC-3') that generated 377- and 401-bp fragments, respectively. The GenBank accession numbers of bovine *BMP15 *and *GDF9 *for PCR analysis are AY572412 and AB058416. All primers were commercially synthesized (Tsukuba Oligo Service, Ibaraki, Japan). The program comprised an initial denaturation step at 95°C for 30 sec, annealing at 58°C for 30 sec, and extension at 72°C for 1 min. Each PCR was performed for 30 cycles for each sample. PCR products were analyzed using agarose gel electrophoresis and visualized with Gel Star Nucleic Acid Gel Stain (Cambrex Bio Science Rockland, Inc, USA). Bovine glyceraldehyde-3-phosphate dehydrogenase (*GAPDH*) was used as a control for PCR. To clarify the contamination of oocytes, PCR analysis of germ cell markers *VASA *and *ZAR1 *was performed in a cDNA template of cumulus and mural granulosa cells. Primer sequences of *VASA *(5'- CAATTCGACAAATAGTACAAGG-3' and 5'- CAAGAACTGGGCACTTTCC-3') and *ZAR1 *(5'- GGAGCTGGGCAAGGAGCG-3' and 5'- TTTGAAGCTGAAAGTGCTGTCAC-3') were used from a previous report [18].

### QPCR

QPCR analyses for the gene expression of *BMP15*, *GDF9 *and *FSHR *in ovarian cells were carried out by the SYBR Green assay as previously reported [29,30]. The thermal cycling conditions included initial sample incubation at 50°C for 2 min and at 95°C for 10 min, followed by 40 cycles at 95°C for 15 sec and at 60°C for 1 min. The cycle threshold values (C_T_) indicated the quantity of the target gene in each sample, and the sequence of the target gene was determined in real time using an Mx3000P QPCR system (Stratagene, La Jolla, CA, USA). Standard curves were generated for each gene by serial dilution of pGEM-cloning vectors containing *BMP15*, *GDF9*, and *GAPDH *cDNAs to quantify the amplified products. Real-time RT-PCR was performed using primers (BMP15: 5'-ATCATGCCATCATCCAGAACC-3' and 5'- TAAGGGACACAGGAAGGCTGA = 3', GDF9: 5'- AGCGCCCTCACTGCTTCTATAT-3' and 5'- TTCCTTTTAGGGTGGAGGGAA-3', FSHR: 5'-AATCTACCTGCTGCTCATAGCCTC-3' and 5'- TTTGCCAGTCGATGGCATAG-3') that generated 72-, 80- and 76-bp fragments. The GenBank accession number of bovine *FSHR *is NM174061.

### In situ hybridization

Digoxigenin (DIG)-labeled bovine *BMP15 *and *GDF9 *sense- and antisense-complementary RNA probes were synthesized as described in a previous study [31]. The PCR products of *BMP15 *and *GDF9 *in the previous RT-PCR section were used as the templates for the RNA probe. Embedded ovaries were sectioned into 7-μm thick slices. *In situ *hybridization was performed using an automated processing machine (Ventana HX System Discovery) with RiboMapKit, BlueMapKit, and AmpMapKit (Ventana, Tucson, AZ, USA) according to the manufacturer's instructions. The sections were fixed and treated with protease after deparaffinization. DIG-labeled probes diluted in RiboHybe hybridization solution (Ventana) were added to each section and hybridized at 61°C for 6 hr. Following hybridization, the sections were washed three times in RiboWash (Ventana) at 65°C for 6 min and post-fixed in RiboFix (Ventana) at 37°C for 10 min. To detect the hybridization signals, the sections were incubated with polyclonal rabbit-anti-DIG/HRP conjugate (Dako Cytomation, Glostrup, Denmark) at 37°C for 30 min. AmpMapKit (Ventana) was used to sensitize the hybridization signals. BlueMapKit (Ventana) with NBT/BCIP was used to color the hybridized signals blue. Counterstaining was performed with Nuclear Fast Red (Ventana). After preparation, the sections were mounted and observed with a Nikon ECLIPSE E800 photomicroscope (Nikon, Tokyo, Japan).

### Production of bovine BMP15 and GDF9 antibody

Recombinant BMP15 and GDF9 were expressed by using a cell-free system (Rapid Translation Systems, Roche Diagnostics, Basel, Switzerland). Bovine cDNAs encoding the mature protein regions of BMP15 and GDF9 were cloned by RT-PCR with high-fidelity DNA polymerase (*Pfu *polymerase, Stratagene, La Jolla, CA, US). Amino termini of mature protein regions were predicted based on the consensus motif R-X-X-R as a target of subtilisin-like proteases.

Cloned sequences were subcloned to the pIVEX 2.4d expression vector (Roche). Protein expression was carried out using *E.coli *lysate reagent (RTS Proteomaster HY Kit, Roche) according to the manufacturer's instructions. The RTS reaction chamber used in the present study contained 1 ml of reaction mixture and 11 ml of feeding solution. After 24 hr of incubation, 1 ml of reaction mixture was harvested from the chamber, solubilized with 4 ml of 8 M urea solution, and centrifuged at 22000 × g for 10 min at 4°C to collect the supernatant. Recombinant proteins were purified from the supernatant using a nickel sepharose affinity gel (GE Healthcare, Buckinghamshire, UK) in the presence of 6 M urea, based on the chromatography. Anti-BMP15 and GDF9 antisera were generated in rabbits. The rabbits were bled prior to immunization to obtain preimmunoserum. They were initially inoculated with 300 μg of the antigen in Freund's complete adjuvant. Three weeks after initial immunization, the rabbits were further inoculated with 150 μg of the antigen in Freund's incomplete adjuvant. Animals were given three booster injections at two-week intervals. The titer of antiserum was monitored by ELISA. Two weeks after the third booster injection, animals were exsanguinated to collect antiserum.

### Western blot analysis

To confirm the immunoreactive property of custom anti-BMP15 and GDF9 antisera, recombinant BMP15 and GDF9 were used for Western Blot analysis. Samples (100ng each) were separated by sodium dodecyl sulfate-polyacrylamide electrophoresis and electrophoretically transferred onto a polyvinylidene difluoride membrane [32]. Western blotting was performed using the method of Towbin *et al. *in Tris-buffered saline (TBS) [33]. The membrane was blocked in 10% skim milk overnight, incubated with either anti-BMP15 or -GDF9 antisera in 1% skim milk in TBS for 1 hr at room temperature and then washed with TBS containing 0.05% Tween20 (TBST). It was then incubated with anti-rabbit IgG conjugated with alkaline phosphatase (Sigma, diluted 1:3000) for 1 hr at room temperature and then washed with TBST. Immunopositive bands were detected using AP substrate kit (BioRad, Hercules, CA, USA).

### Immunohistochemistry

Immunohistochemistry was performed using an automated processing machine with the RiboMapKit and DABMapKit reagents (Ventana). The sections were incubated with antiserum or pre-immunoserum at a dilution of 1:100 in Ab Diluent (Ventana) for 2 hr. For the first antibody, not only custom antibody, but also commercial antibody; anti-humanBMP15 antibody (Abjent, CA, US) and anti-mouse GDF9 antibody (Santa Cruz CA, USA) were used. The sections were then washed and incubated with anti-rabbit IgG-Biotin (Sigma) for 1 hr. Immunoreactive signals were detected using streptavidin-HRP and diaminobenzidine (DABMapKit, Ventana). After preparation, the sections were observed with a Nikon ECLIPSE E800 photomicroscope (Nikon, Tokyo, Japan).

### Statistical analysis

Rates of antral follicles and COCs were analyzed by a Chi-square test. A *t*-test was used to compare the results of QPCR quantification. The experiments were carried out in at least three replicates. The results quantifying copy numbers of *BMP15 *and *GDF9 *mRNA are expressed as the mean of the ratio of *BMP15 *or *GDF9 *to *GAPDH*. Statistical significance was set at *P *< 0.05.

## Results

**Figure 1:**
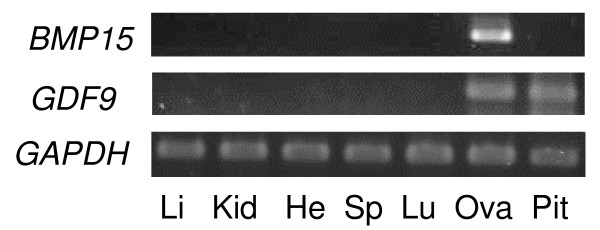
**mRNA expression of *BMP15 *and *GDF9 *in bovine tissues by RT-PCR**. Total RNAs were extracted from liver (Li), kidney (Kid), heart (He), spleen (Sp), lung, (Lu) ovarian (Ov), and pituitary (Pit) tissues. Signal produced by BMP15 primers (377-bp) was only detected in the ovarian tissues. Signals produced by GDF9 primers (401-bp) were detected in the ovarian and pituitary tissues.

**Figure 2:**
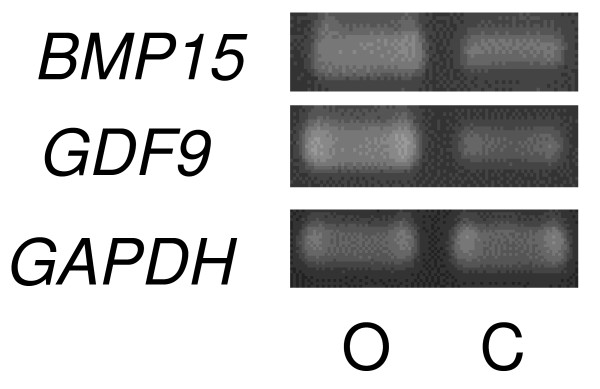
**mRNA expression of *BMP15 *and *GDF9 *in follicles by RT-PCR**. Total RNAs were extracted from oocytes (O) and cumulus cells (C). Signal produced by BMP15 primers (377-bp) and GDF9 primers (401-bp) were detected in the oocytes and cumulus cells.

*BMP15 *transcripts were detected in ovarian tissues, but were not detected in any other tissues examined by RT-PCR. In contrast, *GDF9 *transcripts were detected not only in the ovarian tissues but also in the pituitary tissues, but were not detected in the liver, kidney, heart, spleen or lung tissues (Figure 1). In both oocytes and cumulus cells collected from adult cows, *BMP15 *and *GDF9 *transcripts were detected (Figure 2).

**Figure 3:**
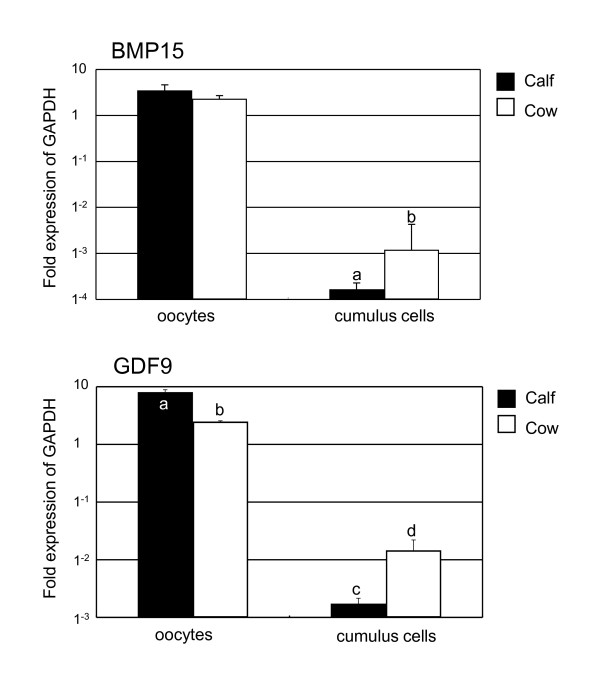
**QPCR analysis of *BMP15 *and *GDF9 *mRNA in the cells of ovaries derived from calves or cows**. Total RNA was extracted from oocytes, cumulus cells and mural granulosa cells. The expression of these mRNAs was normalized to the expression of *GAPDH *measured in the same RNA preparation. Results of three independent experiments are summarized and expressed as the mean ± SEM. Different letters above the bars indicate significant differences (*P *< 0.05).

**Figure 4:**
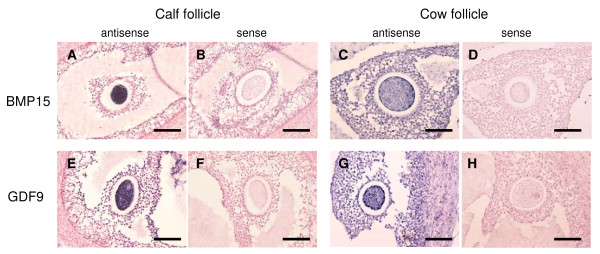
**Localization of *BMP15 *and *GDF9 *mRNA in antral follicles**. *BMP15 *(A-D) and *GDF9 *(E-H) mRNA were detected using *in situ *hybridization. Positive cells are stained blue. The sections of calf (A, B, E, F) and adult (C, D, G, H) ovaries were individually hybridized with DIG-labeled anti-sense (A, C, E, G) and sense (negative control; B, D, F, H) RNA probes. The scale bar is 100 μm.

**Figure 5:**
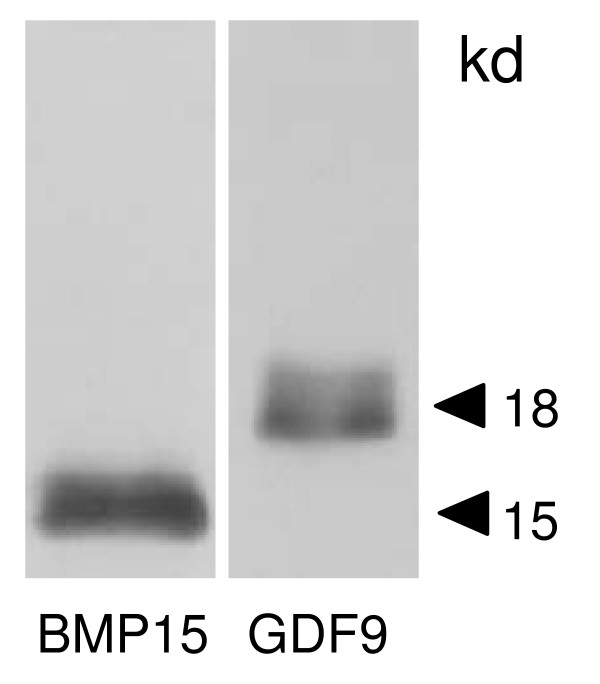
**Western blot analysis of recombinant His-tag fusion bBMP15 and GDF9 protein**. Recombinant proteins were loaded onto separate lanes and separated by SDS-PAGE. Specific proteins were detected by using custom BMP15 and GDF9 antibody.

**Figure 6:**
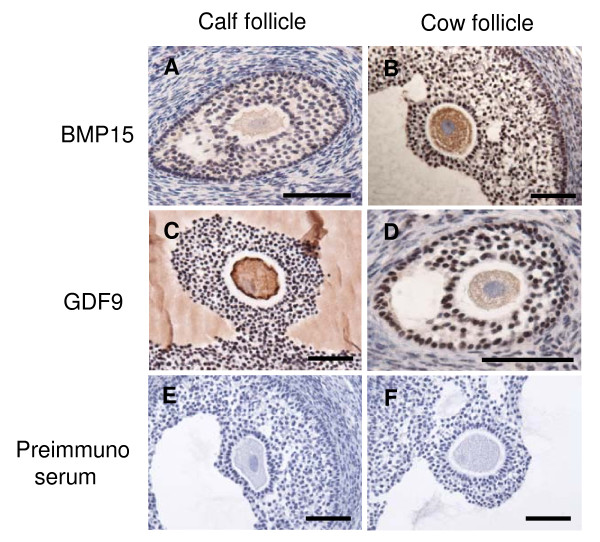
**Localization of BMP15 and GDF9 in calf and cow follicles**. BMP15 and GDF9 immunoreactivity found in oocyte and cumulus cells of calf (A, C) and cow (B, D) follicles. BMP15 and GDF9 proteins were staining brown by immunostain using BMP15 and GDF9 antibody (A,B and C,D) or preimmuno serum with diluent (negative control; E, F). The scale bar is 100 μm.

The amount of class A and B COCs collected from calf ovaries (59.3%, n = 59) was significant less than that from adult ovaries (77.0%, n = 87) (P < 0.05). The germ cell marker *VASA *and *ZAR1 *transcripts were detected in the cDNA template of the oocytes, but not in the cDNA template of cumulus cells (data not shown). Therefore, it was confirmed that the cDNA template of cumulus cells were not contaminated with the cDNA template of oocytes. QPCR detected significantly higher expression of *BMP15 *and *GDF9 *in cumulus cells of adult ovaries than in calf ovaries (Figure 3). *GDF9 *expression in the oocytes of calf ovaries was significantly higher than in adult ovaries. In contrast, *BMP15 *expression in the oocytes of calf and adult ovaries was not significantly different. These results were also ascertained by *in situ *hybridization (Figure 4). RNA probes, DIG-labeled *BMP15 *and *GDF9 *anti-sense RNAs specifically detected the mRNA transcript in calf and adult ovaries (Figure 3A, C, E and 3G), while the sense probe detected no significant signal (Figure 3B, D, F and 3H). In the antral follicles of calf ovaries, *BMP15 *and *GDF9 *mRNAs were strongly detected in the oocytes (Figure 3A and 3E) and were weakly detected in the cumulus cells. In the antral follicles of adult ovaries, *BMP15 *and *GDF9 *mRNAs were detected in the cumulus cells as well as in the oocytes of antral follicles (Figure 3C and 3G). *GDF9 *mRNA was also detected in some mural granulosa cells and in a few theca cells (Figure 3G). The BMP15 and GDF9 recombinant proteins with 6xHis tag were produced by using a cell free system as approximately 15 and 18 kDa. Subsequently generated antisera against either BMP15 or GDF9 reacted with immunized antigens in Western blot analysis (Figure 5). BMP15 and GDF9 localization was determined by immunohistochemistry using both custom and commercial antisera in calf and cow ovaries (Figure 6). Immunopositive signals of BMP15 and GDF9 were detected in the oocytes and cumulus cells of follicles in both calf (Figure 6A and 6C) and cow (Figure 6B and 6D) ovaries. There was no difference in the localization of immunopositive signals in sections incubated with either commercial or in house antisera. The localization of BMP15 and GDF9 protein coincided with that of *BMP15 *and *GDF9 *mRNA. In contrast, the additions of pre-immuno sera, instead of BMP15 and GDF9 antisera, made no immunoreactive signals (Figure 6E and 6F) in the follicles.

**Figure 7:**
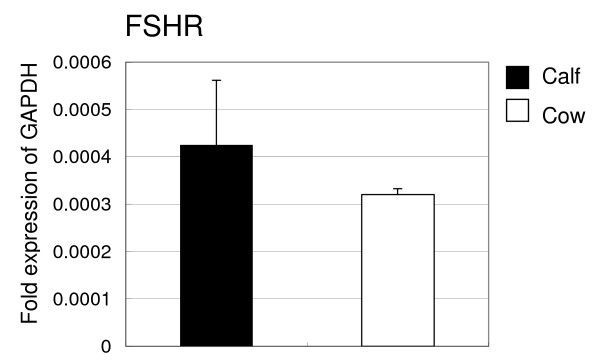
**QPCR analysis of *FSHR *mRNA in the cells of ovaries derived from calves or cows**. Total RNA was extracted from cumulus cells and mural granulosa cells. The expression of these mRNAs was normalized to the expression of *GAPDH *measured in the same RNA preparation. Results of three independent experiments are summarized and expressed as the mean ± SEM. Different letters above the bars indicate significant differences (*P *< 0.05).

QPCR revealed that the intensity of *FSHR *in the cumulus cells of calf and adult ovaries was not significantly different (Figure 7).

## Discussion

In this research, our data showed the existence of bovine *BMP15 *and *GDF9 *mRNA and protein expression, not only in the oocytes but also in follicular somatic cells. Also we showed new differences in mRNA expression patterns of *BMP15 *and *GDF9 *in oocytes and cumulus cells between calf and cow ovaries.

There was no difference in the amount of intra oocyte *BMP15 *mRNA between calf and cow, and was smaller in calf cumulus cells than in cow cumulus cells. Thus, it is predicted that the amount of BMP15 in follicles is greater in cows than in calves. The lower developmental competence of calf oocytes may be partially explained by a deficiency of BMP15 in cumulus cells. It was showed the possibility which additional BMP15 in IVM improve the developmental competence of calf oocytes.

*GDF9 *mRNA expression in cumulus cells was detected by QPCR and *in situ *hybridization even though at a low level. In previous reports, *GDF9 *mRNA expression was detected in oocytes, but not in bovine cumulus and mural granulosa cells by RT-PCR [34] or by *in situ *hybridization [35]. Our results differ from these previous reports. This difference in expression may be related to the status of follicle atresia or to the estrous cycle. The amount of *GDF9 *gene expression was smaller in calf cumulus cells than in cow cumulus cells, same as the amount of *BMP15*. The two factors are thought to have synergistic effect to proliferate or regulate granulosa cells [2,7,8]. There is a possibility that the smaller expression of both BMP15 and GDF9 in cumulus cells of calves affect function of granulosa cells. The amount of *GDF9 *gene expression was greater in calf oocytes, than in cow oocytes, unlike in the cumulus cells. It is required further study to clarify relationship between the low developmental competence of calves and GDF9.

BMP15 inhibits FSH-induced granulosa cell differentiation through down-regulation of FSH receptor (FSHR) expression in rat granulosa cells [11]. In our results, although QPCR revealed that the intensity of *BMP15 *expression in the cumulus cells of adult ovaries was significantly higher than that of calf ovaries, the intensity of *FSHR *in cumulus cells of calf and adult ovaries was not significantly different. In mutant sheep, the FSHR binding assay showed no difference in FSH responsiveness in granulosa cells between animals heterozygous for BMP15 mutation, whose phenotype is a higher ovulation rate, and wild-type [36]. Whether ruminant BMP15 inhibits *FSHR *gene expression and FSHR activity needs further investigation. Previously, we reported that *FSHR *mRNA expression was greater in bovine largest (10.7+/-0.7mm) and healthy follicles than in second-largest (7.8+/-0.2 mm) and atretic follicles [30]. However, our results could not clarify the relationship between the developmental competence of oocytes and *FSHR *mRNA expression in the cumulus cells.

When comparing rodents with an incomplete estrus cycle (mice, rats) to other animals with a complete estrous cycle (humans, goats pigs and cows), GDF9 and BMP15 appear in different tissues. In rodents, BMP15 and GDF9 expressed exclusively in oocytes [1,13,37,38]. However, in other species, including humans [39], goats [5], pigs [3] and cows (this study), BMP15 and GDF9 were expressed in cumulus cells as well as in oocytes. BMP15 and GDF9 have somewhat different roles between rodents and ruminants: for example, in the phenotypes of null mutations [12,14,16] and the ability to regulate ovulation rates [12,14,16,40-42]. It was speculated that these species-specific functional differences between monoovulatory human and sheep and polyovulatory mouse were attributable to the timing of processing of the BMP15 proprotein into a functionally mature BMP15 [43]. Although the biological significance of BMP15 expression in follicular somatic cells has not been fully elucidated, previous reports and the results of the present study indicate the possibility that BMP15 in somatic cells as well as in oocytes may regulate the selection of follicle and/or ovulation in species with a complete estrus cycle.

Differences in BMP15 expression between rodents and cows were also detected in the pituitary. In mice, *BMP15 *is expressed in the pituitary [44,45]. It was hypothesized that BMP15 could play a physiological role in the monotropic rise of FSH secretion by the pituitary during the estrous and menstrual cycle [45]. However, *BMP15 *was not expressed in the bovine pituitary (Figure 1). In contrast, *GDF9 *was detected in rodents and human pituitary [46]. Our results also revealed that *GDF9 *mRNA was expressed in the bovine pituitary (Figure 1). The physiological role of bovine GDF9 in the pituitary remains to be determined.

In conclusion, this study demonstrated, for the first time, that the intensity of expression of transcripts encoding *GDF9 *and *BMP15 *differed between calf and adult ovaries. Our results suggest that the lower developmental competence in calf oocytes is associated with immature expression of BMP15/GDF9 in an intrafollicular environment.

## Conclusions

We characterized the gene expression of BMP15 and GDF9 in calf and adult bovine oocytes and cumulus cells using QPCR and *in situ *hybridization. The expression of *BMP15 *and *GDF9 *in cumulus cells of adult ovaries was significantly higher than that in calf ovaries. *GDF9 *expression in the oocytes of calf ovaries was significantly higher than that of the adult ovaries. The localization of gene and protein expression was ascertained by *in situ *hybridization and immunohistochemintry. We suggest the possibility that the lower developmental competence of calf oocytes when compared to that of cow oocytes, is related to the different expression of *BMP15 *and/or *GDF9 *in oocyte and cumulus cells.

## Competing interests

The authors declare that they have no competing interests.

## Authors' contributions

MH participated in the design of the study, collected the materials, carried out all experiments and drafted the manuscript. KK, KU, KGH and TT collected the materials and helped to carry out RT-PCR, QPCR and *in situ *hybridyzation. KU and TT also helped to draft the manuscript. All authors read and approved the final manuscript.
